# Modification of improved-genome editing via oviductal nucleic acids delivery (*i*-GONAD)-mediated knock-in in rats

**DOI:** 10.1186/s12896-021-00723-5

**Published:** 2021-11-01

**Authors:** Takuya Aoshima, Yukari Kobayashi, Hisayoshi Takagi, Kenta Iijima, Masahiro Sato, Shuji Takabayashi

**Affiliations:** 1 Laboratory Animal Facilities and Services, Preeminent Medical Photonics Education and Research Center, Hamamatsu University School of Medicine, 1-20-1 Handayama, Higashi-ku, Hamamatsu, Shizuoka 431-3192 Japan; 2 Department of Genome Medicine, National Center for Child Health and Development, 2-10-1 Okura, Setagaya, Tokyo 157-8535 Japan

**Keywords:** Genome editing, *i*-GONAD; rat, In vivo electroporation, CRISPR/Cas9, Tyrosinase

## Abstract

**Background:**

Improved genome-editing via oviductal nucleic acids delivery (*i*-GONAD) is a new technology that facilitates in situ genome-editing of mammalian zygotes exiting the oviductal lumen. The *i*-GONAD technology has been developed for use in mice, rats, and hamsters; however, oligonucleotide (ODN)-based knock-in (KI) is more inefficient in rats than mice. To improve the efficiency of *i*-GONAD in rats we examined KI efficiency using three guide RNAs (gRNA), crRNA1, crRNA2 and crRNA3. These gRNAs recognize different portions of the target locus, but also overlap each other in the target locus. We also examined the effects of commercially available KI -enhancing drugs (including SCR7, L755,507, RS-1, and HDR enhancer) on *i*-GONAD-mediated KI efficiency.

**Results:**

The KI efficiency in rat fetuses generated after *i*-GONAD with crRNA2 and single-stranded ODN was significantly higher (24%) than crRNA1 (5%; *p* < 0.05) or crRNA3 (0%; *p* < 0.01). The KI efficiency of *i*-GONAD with triple gRNAs was 11%. These findings suggest that KI efficiency largely depends on the type of gRNA used. Furthermore, the KI efficiency drugs, SCR7, L755,507 and HDR enhancer, all of which are known to enhance KI efficiency, increased KI efficiency using the *i*-GONAD with crRNA1 protocol. In contrast, only L755,507 (15 μM) increased KI efficiency using the *i*-GONAD with crRNA2 protocol. None of them were significantly different.

**Conclusions:**

We attempted to improve the KI efficiency of *i*-GONAD in rats. We demonstrated that the choice of gRNA is important for determining KI efficiency and insertion and deletion rates. Some drugs (e.g. SCR7, L755,507 and HDR enhancer) that are known to increase KI efficiency in culture cells were found to be effective in *i*-GONAD in rats, but their effects were limited.

**Supplementary Information:**

The online version contains supplementary material available at 10.1186/s12896-021-00723-5.

## Background

Clustered regularly interspaced short palindromic repeat (CRISPR)/CRISPR-associated protein 9 (Cas9) can induce double-strand breaks (DSBs) in DNA. The repair of the DSB require the cellular machinery, which can be performed by homology-directed repair (HDR), non-homologous end joining (NHEJ) or microhomology-mediated end-joining (MMEJ). In the absence of donor (or template) DNA, these DSBs are repaired via NHEJ, which is error-prone. NHEJ often generates random insertions or deletions (indels) or substitutions of nucleotides at the break site, but not always. If a donor DNA containing single-stranded (ss) sequences or double-stranded sequences with homology to the target region is present, it can be introduced into the DSB site through HDR, which is a cellular mechanism enabling the precise repair of DSBs. The most common form of HDR is homologous recombination (HR), also known as knock-in (KI) in which plasmids, synthetic oligodeoxynucleotides (ODNs), or large DNA fragments (up to 7.4 kb) are frequently used as DNA donors. HDR facilitates the creation of transgenic animals with precise KI alleles or those carrying conditional alleles (floxed alleles). The coinjection of specific donor plasmids [1, 2] or ssODN donors [3–5] is typically used in the creation of KI animals. Generally, the HDR-mediated KI has been reported to occur less frequently than NHEJ-mediated indels events, so-called knock-out (KO) [6].

Recent advances in gene editing technology (as exemplified by the CRISPR/Cas9 system) have enabled the efficient production of genetically modified (GM) animals including mice, which has accelerated the discovery of gene functions and the creation of animal models of human disease. GM animals have mainly been produced by the microinjection of genome editing components into zygotes [7–11] or the in vitro electroporation (EP) of zygotes in the presence of genome editing components [12–14]. However, these procedures need zygotes isolated from pregnant females (or those obtained through in vitro fertilization), the temporal cultivation of treated zygotes, and the transfer of genome-edited embryos to the reproductive tracts of pseudopregnant females for further development, all of which are laborious and time-consuming. Furthermore, microinjection procedures require expensive micromanipulator systems and highly skilled operators. To overcome this, *G*enome editing via* O*viductal *N*ucleic *A*cids *D*elivery (GONAD) was first developed by Takahashi et al. as a novel method for creating GM animals [15]. This involves the injection of genome editing components into the lumen of oviducts of pregnant females at embryonic day (E) 1.4 (corresponding to the 2-cell stage) under observation using a dissecting microscope, and the subsequent in vivo EP of the entire oviduct to facilitate the uptake of genome editing components by 2-cell embryos. Later, it was found that GM animals (mice and rats) could be created by performing GONAD in late 1-cell stage embryos (at E0.7) in the “improved-GONAD (*i*-GONAD)” technique [16, 17]. GONAD/*i*-GONAD do not require the ex vivo handling of preimplantation embryos, micromanipulator systems, microinjections and egg transfers, or the preparation of pseudopregnant females and vasectomized males. Instead, they only require a square pulse-generating electroporator. We previously used *i*-GONAD to produce GM rats and KO/KI rats [18]. Unfortunately, the success rate of KI rats was lower than that of KI mice (5% vs. 60%, respectively) when ssODNs were used as KI donors [17, 18]. The laboratory rat has long been used in many studies to model a specified trait of human diseases. Recent studies with GM rats have revealed that observed disease phenotypes are often more similar than mouse models to those of humans [19]. So, it is important to improve the success rate of KI rats.

In this study, we attempted to improve the KI efficiency in rats by using three guide RNAs (gRNAs) that recognize different but similar portions of a target locus (the tyrosinase gene, *Tyr*) or by employing reagents known to be effective at increasing KI such as SCR7, Alt-R^Ⓡ^ Cas9 Electroporation Enhancer (hereafter called “EP Enhancer”), azidothymidine (hereafter called “AZT”), L755,507 (hereafter called “L755”), RAD51-stimulatory compound 1 (hereafter called “RS-1”), and Alt-R^Ⓡ^ HDR Enhancer (hereafter called “HDR Enhancer”) [20–27].

## Results

### *i*-GONAD-mediated KI using three gRNAs

**Fig. 1:**
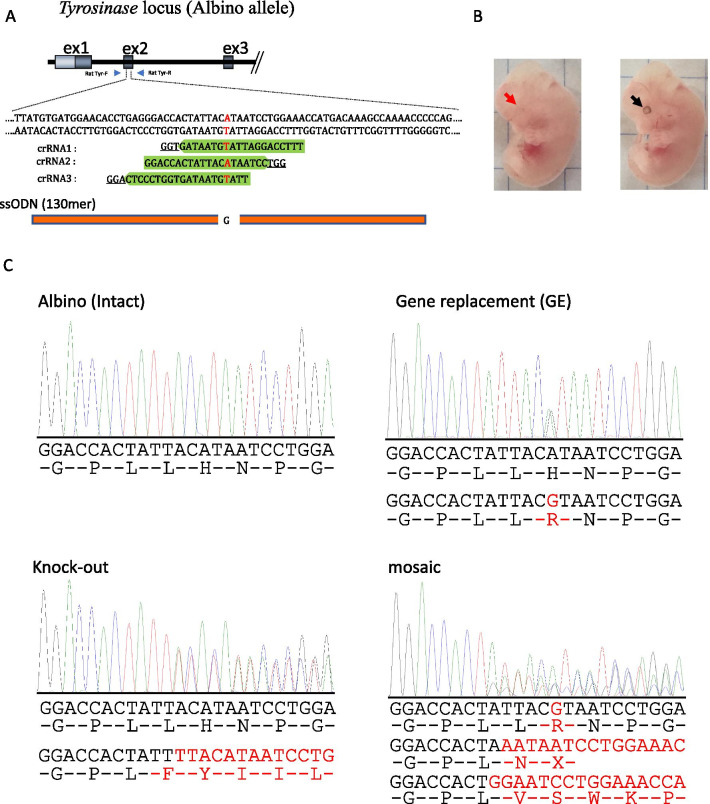
**a** Schematic of the mutated *Tyr* locus. The target sequence (exon 2 of *Tyr*) recognized by crRNA1, 2, and 3 is shown in green. The PAM sequences are underlined. ssODN (containing wild-type nucleotide “G” that corresponds to mutated nucleotide “A”) is shown in orange below the target sequence. Nucleotide “A/T” marked in red is the mutation causative of the albino phenotype. Primers F and R used for amplification of the region spanning the target sequence are shown below *Tyr*. **b** Mid-gestational fetuses obtained from *i*-GONAD-treated females. Left: Albino fetus exhibiting depigmented eyes (red arrow). Right: Fetus exhibiting pigmented eyes (black arrow), reflecting successful KI of ssODN into the *Tyr* locus of albino rats. **c** Direct sequencing of PCR products derived from the albino (intact) fetus, KI fetus, KO fetus, and mosaic fetus

Because the rate of indel production caused by CRISPR/Cas9 is known to be largely affected by the type of gRNA used [28, 29], we first examined the KI efficiency in rat fetuses when *i*-GONAD was performed using three gRNAs (crRNA1, 2, and 3) designed to target rat *Tyr* (exon 2) with a G to A mutation by KI (Fig. 1a). *i*-GONAD was performed in the presence of Cas9 protein, gRNA (crRNA and tracrRNA), and ssODN on E0.7 of pregnancy. Developing fetuses were dissected and examined on E14.5-E16.5, and their genomic DNA was isolated. Successful KI was easily discernible by determining the eye color in *i*-GONAD-treated fetuses and direct sequencing of PCR products.

**Fig. 2:**
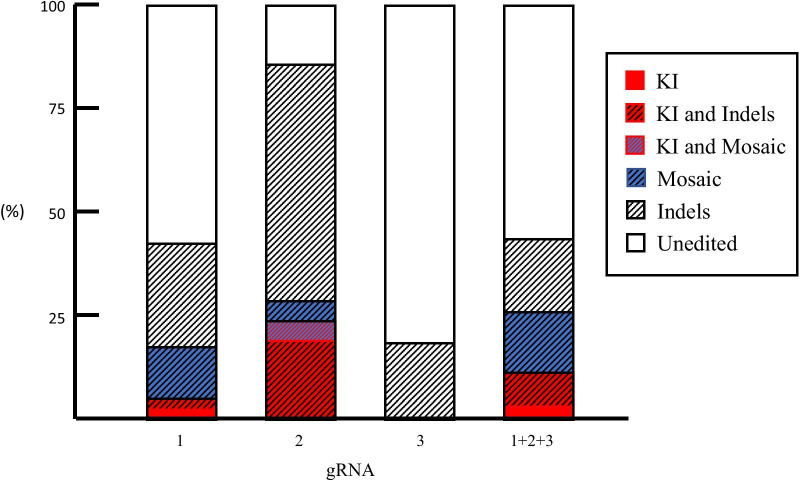
The KI efficiency, indels efficiency, mosaic mutations efficiency, and unedited efficiency are shown as a sum total of 100%. Abbreviations: 1, *i*-GONAD using crRNA1; 2, *i*-GONAD using crRNA2; 3, *i*-GONAD using crRNA3; 1 + 2 + 3, *i*-GONAD using a mixture of three gRNAs. KI rates are shown in red square.KI and indels rates are shown as black shaded lines on red background. Indels rates are shown as black shaded lines. Mosaic mutations rates are shown as black shaded lines on blue background. KI and mosaic rete are shown as blue shaded lines on red background. Unedited rates are shown in white square

The results are shown in Table 1, Fig. 2 and Additional file 2: Fig. S1. The KI efficiency varied largely, depending on the type of gRNA used. The highest efficiency was observed when *i*-GONAD using crRNA2 was carried out, with five (24%) of 21 fetuses exhibiting pigmented eyes (Table 1, Fig. 1b, right; Fig. 2; Additional file 2: Fig. S1). Direct sequencing of PCR products derived from these five samples demonstrated that all had at least one KI allele (Fig. 1c, top right) and also had indels. Sixteen (76%) still had albino eyes (Fig. 1b, left), but sequencing revealed one or more alleles with indels (Fig. 1c, bottom left), except for three albino fetuses (14%) with no mutation (Fig. 1c, top left; Table 1; Fig. 2). A total of 18 (86%) had indels (Table 1 and Additional file 2: Fig. S1). Two (10%) were identified as mosaic individuals by direct sequencing because one had one KI allele plus two alleles with indels (Fig. 1c, bottom right; Table 1) and the other had one intact allele plus two alleles with indels. These fetuses with indel or mosaic alleles also showed the highest efficiency among three gRNAs examined (Additional file 2: Fig. S1; Table 1). *i*-GONAD with crRNA1 showed the second highest efficiency, with two (5%) of 40 fetuses exhibiting pigmented eyes (Additional file 2: Fig. S1; Table 1), and fetuses with indel or mosaic alleles seen in 40% and 13%, respectively (Table 1; Fig. 2). crRNA3 was associated with the lowest efficiency, resulting in no fetuses with a KI allele (0/38; Additional file 2: Fig. S1; Table 1), and fetuses with indel or mosaic alleles seen in 18% and 0%, respectively. Notably, KI and indel efficiencies in samples with crRNA2 were significantly higher than those in samples with crRNA1 or crRNA3 (*p* < 0.05; Additional file 2: Fig. S1; Table 1).

**Table 1:** Summary of *i*-GONAD using knock-in enhancers

crRNA used	Enhancers^1^	No. of pregnant rats/no. of treated rats	Total number of fetuses obtained	No. of fetuses or pups carrying pigmented eyes (successful KI [%])	No. of fetuses showing indels (%)	No. of fetuses showing mosaic mutations (%)	No. of fetuses showing no mutation (%)
1	–	4/7	40	2 (5)	16 (40)	5 (13)	23 (58)
	1 µM SCR7	6/6	27	2 (7)	11 (41)	6 (22)	16 (59)
	10 µM SCR7	4/4	23	2 (9)	10 (43)	5 (22)	13 (57)
	100 µM SCR7	3/3	32	2 (6)	16 (50)	4 (13)	15 (47)
	4 µM EP Enhancer	2/4	12	0 (0)	4 (33)	1 (8)	8 (67)
	30 µM AZT	4/4	21	0 (0)	10 (48)	1 (5)	11 (52)
	5 µM L755	4/4	26	3 (12)	9 (35)	4 (15)	17 (65)
	15 µM L755	5/6	33	4 (12)	14 (42)	2 (6)	17 (52)
	15 µM RS-1	6/9	25	2 (8)	12 (48)	2 (8)	13 (52)
	30 µM HDR Enhancer	4/4	28	3 (11)	16 (57)	8 (29)	12 (43)
	Mixture of four enhancers^2^	3/3	21	2 (10)	11 (52)	3 (14)	9 (43)
2	–	4/4	21	5 (24)	18 (86)	2 (10)	3 (14)
	10 µM SCR7	3/3	30	5 (17)	16 (53)	4 (13)	12 (40)
	5 µM L755	3/4	18	3 (17)	9 (50)	2 (11)	8 (44)
	15 µM L755	3/3	20	6 (30)	9 (45)	3 (15)	6 (30)
	30 µM HDR Enhancer	4/4	22	3 (14)^2^	10 (45)	2 (9)	11 (50)
3	–	5/5	38	0 (0)	7 (18)	0 (0)	31 (82)
1 + 2 + 3	–	8/9	62	7 (11)	25 (40)	9 (15)	35 (56)

^1^EP Enhancer, Alt-R® Cas9 Electroporation Enhancer; AZT, azidothymidine; L755, L755,507; RS-1, RAD51-stimulatory compound 1; HDR Enhancer, Alt-R® HDR Enhancer

^2^Mixture of 10 µM SCR7, 5 µM L755, 15 µM RS-1, and 30 µM HDR Enhancer

–in vivo EP in the absence of any reagent

We next checked whether the KI efficiency was improved with a mixture of three gRNAs. We obtained 62 fetuses from eight pregnant rats, of which seven (11%) had pigmented eyes (Fig. 2; Additional file 2: Fig. S1; Table 1). This efficiency was intermediate between *i*-GONAD with crRNA2 alone and that with crRNA1 alone. Furthermore, the rates of fetuses with indel or mosaic alleles were intermediate between *i*-GONAD with crRNA2 alone and that with crRNA1 alone. These findings suggest that the choice of gRNA is an important factor affecting KI efficiency.

### Effects of KI-enhancing drugs on the rate of *i*-GONAD-mediated KI

We next examined whether the addition of commercially available drugs known to increase KI efficiency would do so in rats. Drug properties are described in Additional file 1: Table S1. *i*-GONAD using a solution containing ribonucleoprotein (RNP) complex (consisting of Cas9 protein and gRNA), ssODN, and various amounts of drugs (1, 10, and 100 μM for SCR7, 4 μM for EP Enhancer, 30 μM for AZT, 5 and 15 μM for L755, 15 μM for RS-1, or 30 μM for HDR Enhancer) was performed on E0.7 of pregnancy. crRNA1 was used as the second most effective gRNA for *i*-GONAD-mediated KI. The enhancers that were effective in crRNA1 were also examined in crRNA2 (Table 1). L755 exhibited maximal effects at 5–15 μM. Three (12%) of 26 fetuses showed pigmented eyes and molecular evidence for successful KI when 5 μM L755 was used for *i*-GONAD, and four (12%) of 33 fetuses had pigmented eyes when *i*-GONAD with 15 μM of L755 was performed (Table 1). These KI efficiencies (12% for each group) were 2.4-fold higher than that (5%) obtained with *i*-GONAD using crRNA1 alone. Additionally, KI efficiencies had increased slightly when 10 μM SCR7 and 30 μM HDR Enhancer was used for *i*-GONAD. *i*-GONAD using HDR Enhancer had an efficiency of 11% (3/28), which was the second highest efficiency (Table 1). Unfortunately, these treatments failed to produce significant differences between the drug-treated and untreated groups. For example, *i*-GONAD with SCR7 at 1, 10, or 100 μM resulted in KI efficiencies of 7%, 9%, and 6%, respectively, while *i*-GONAD using 15 μM of RS-1 resulted in an efficiency of 8% (Table 1). Neither EP Enhancer nor AZT improved *i*-GONAD-mediated KI (Table 1). *i*-GONAD in the presence of RNP (containing crRNA1 and Cas9), ssODN, 10 µM of SCR7, 5 µM of L755, 15 µM of RS-1, and 30 µM of HDR Enhancer resulted in two pigmented fetuses (10%) out of 21 fetuses (Table 1), suggesting no appreciable synergistic effects of the drugs in improving *i*-GONAD-mediated KI.

Similar findings were observed with crRNA2. The addition of 15 µM of L755 caused eye pigmentation in six (30%) of 20 fetuses tested, which was 1.25-fold higher than that seen from *i*-GONAD with crRNA2 alone (24%). However, the addition of SCR7 (or HDR Enhancer) had no effect on KI efficiency (Table 1).

Moreover, no decrease in pregnancy rate was observed with the addition of any drugs (Table 1).

## Discussion

According to Raveux et al. [30], ssODN-mediated KI efficiency varied from 0 to 40%, depending on the type of Cas9, injection site, length of homology arm, and gRNA binding site. The nucleotide composition of a target sequence is one of the most important factors determining KI and KO efficiency. For example, Graf et al. [31] screened published gRNA activity datasets and demonstrated that TT- and GCC-motifs located in the four PAM-proximal bases of the targeting sequence (the efficiency-modulating sequence) were sufficient to block CRISPR/Cas9-mediated KI. Moreover, the presence of these motifs in gRNAs resulted in a tenfold reduction of gene KO frequencies. We observed no such inhibitory sequences in our gRNAs (Fig. 1A).

According to CHOPCHOP predictions [32], our three gRNAs exhibited high efficiencies, ranging from 51.18 to 56.50%. The KI efficiency in fetuses generated after *i*-GONAD with crRNA2 was significantly higher (24%) than that obtained after *i*-GONAD with crRNA1 (5%; *p* < 00.5) or crRNA3 (0%; *p* < 0.01) (Fig. 2; Table 1), but was still lower than expected. in vitro genome editing efficiency is known to vary, with predicted efficiencies typically higher than observed levels [8, 33, 34]. However, as shown in Fig. 1A, the three gRNAs used here recognize the *Tyr* sequence containing the point mutation (G to A) and overlap with each other. Therefore, in this context, the discrepancy between predicted and actual efficiencies was unexpected. Jang et al. [35] microinjected overlapping gRNAs (sharing at least 5 bp of the target site), *Cas9* mRNA, and ssODN into 1-cell-stage zygotes for KI mouse generation in ssODN-mediated HDR. They obtained *loxP*-inserted floxed mice or those with single nucleotide-substituted alleles with efficiencies of 18–38%, which was higher than seen in the control of non-overlapping gRNAs (8%) [35]. They speculated that the enhanced efficiency resulted from overlapping gRNAs inducing shorter NHEJ-mediated sequence deletions close to the intended mutation site, which potentially favored HDR [36]. Although our three gRNAs overlapped and shared 11 bp of the target site (Fig. 1a), they did not increase the KI efficiency (15%; Fig. 2; Table 1) but were intermediate between results obtained with crRNA2 and crRNA1.

We observed that crRNA2 gave the highest efficiency of indels (87%; Fig. 2) among the three gRNAs tested, followed by crRNA1 (40%) and crRNA3 (20%). These results correlate well with the KI efficiency. We speculate that gRNAs better able to cleave the target sequence are also more efficient at performing KI. Although Jang et al. [35] observed overlapping gRNAs exhibited higher KI efficiency than non-overlapping gRNAs, there was no differences in the rates of NHEJ-mediated DSB repair between overlapping and non-overlapping gRNAs. Inui et al. [37] also reported a weak correlation between DSB frequency and KI efficiency. These discrepancies may be ascribed to the systems used, for example in vivo EP of zygotes vs. microinjection of zygotes. Future work should further examine the correlation between DSB frequency and KI efficiency.

Several drugs have been reported to enhance CRISPR/Cas9-mediated KI efficiency, including SCR7, RS-1, and L755. Of the six commercially available drugs tested in the present study, L755 was found to exhibit maximal effects at 5–15 μM with an efficiency of 12%, when crRNA1 was used as the gRNA (Table 1). However, this was only slightly (2.4-fold) higher than the control without L755 (5%). The amounts of drugs used in our study were based on previous publications [22, 26, 38, 39] which reported enhanced KI efficiency in cultured cells and embryos. It is conceivable that drugs added to the oviductal lumen via *i*-GONAD may not have been efficiently taken up by zygotes. However, Song et al. [26] found that treating rabbit embryos microinjected with *Cas9* mRNA, gRNA, and donor template DNA with 7.5 μM RS-1 significantly enhanced the KI efficiency to 26.1% compared with the control (4.4%). This suggests that small molecules can be easily incorporated into early embryos under natural conditions. It is therefore expected that drugs added to the oviductal lumen upon *i*-GONAD would readily have been taken up by rat zygotes. Although there was no significant difference in rats *i*-GONAD in this study, it is suggested that there may be significant differences when other animal species (e.g., mouse and rabbit) are used.

Although Scr7 and AZT are known to be potent NHEJ inhibitor and NHEJ promoter, respectively, there was no significant difference in indels efficiency for either drug in this study (Table 1). It may be that EP in the oviduct does not allow enough drug to penetrate the fertilized egg. AZT was not found to be repaired by the HDR pathway, suggesting its effectiveness as an HDR inhibitor.

Notably, *i*-GONAD using RNP (containing crRNA2), ssODN, and 5 μM L755 yielded KI fetuses at 1.25-fold higher rates than controls (RNP + ssODN) (30% *vs*. 24%, respectively; Table 1). Thus, enhanced KI efficiency in rats may result from performing *i*-GONAD in combination with KI-enhancing drugs.

## Conclusions

Because ssODN can be utilized without vector cloning, ssODN-based KI appears to be a simple method of generating KI animals and cells. Therefore, means of increasing the KI efficiency are important, especially for the generation of KI animals. KI-enhancing drugs have been reported to increase KI efficiencies in cells and embryos [22, 26, 38, 39], but we found the effects of commercially available KI-enhancing drugs on the improvement of *i*-GONAD-mediated KI mouse production to be marginal. Other improvements to KI could include the chemical modification of ssODNs [5, 40], the controlled timing of CRISPR/Cas9 delivery [41], and the use of a double cut HDR donor flanked by gRNA-PAM sequences and released after CRISPR/Cas9 cleavage [42]. Surprisingly, we showed that the choice of gRNA is important for determining KI efficiency in rats. *i*-GONAD using crRNA2 generated KI rats with an efficiency of 24%, compared with the 0% when crRNA3 was used.

## Methods

### Animals

Young adult male and female Slc:SD (SD) rats were purchased from Japan SLC Inc. (Shizuoka, Japan) at 10–12 or 8–10 weeks of age, respectively. A total of 86 rats were used in this study. All results are shown in Table 1. Randomly selected rats were used in both the control and drugs addition groups. Animals were kept in the animal room which was maintained at 23 ± 3 °C with a 12-h light/12-h dark cycle (lights on at 07:00 h; lights off at 19:00 h). They were housed in a plastic cage with bedding material and given pellet diet and tap water ad libitum. Our animal facility was maintained under clean conditions. In-house monitoring was performed every 3 months using a Monilisa IVA kit (Wakamoto Pharmaceutical Co Ltd, Tokyo, Japan) to detect four major organisms: Sendai virus, mouse hepatitis virus, mycoplasma and Tyzzer’s organism. No infection was detected in any animal room in which the rats used in this study were maintained.

Experiments involving the in vivo transfection of rat preimplantation embryos by *i*-GONAD were accompanied by surgery (exposure of ovaries/oviducts/uteri) and operation/manipulation (DNA injection via the oviductal wall and in vivo EP). All efforts were made to minimize the number of animals used and their suffering.

All animals were handled in strict accordance with good animal practice as defined by the relevant national and/or local animal welfare bodies, and all animal works were approved by the appropriate committee.

This experiment corresponds to a Category C experiment of the Scientists Center for Animal Welfare (SCAW). We did not have a humane endpoint.

### Estrus cycle monitoring and mating

To confirm suitable times for mating, we employed the vaginal smear method to monitor the estrus cycle of female rats, as described previously [18]. At 08:00–09:00 each morning, we collected vaginal smears using a cotton-tipped swab wetted with distilled water by softly rolling against the vaginal wall. The swab was applied to a dry slide, which was air-dried and stained with Giemsa stain solution (Fujifilm Co., Tokyo, Japan) for approximately 30 min at room temperature. The slide was rinsed with tap water, then air-dried before observation at 40–200× magnification under bright field illumination. The stage of the estrus cycle was determined by the presence and the amount of leukocytes, cornified epithelial cells, and nucleated epithelial cells.

Female rats in proestrus phase were mated with male rats by housing them together overnight. The following noon, females with sperm in their vaginal smear were judged as pregnant, designated E0.5, and used for *i*-GONAD experiments.

### Preparation of CRISPR/Cas9 reagents and KI-enhancing drugs

CHOPCHOP software (https://chopchop.cbu.uib.no/) was used to predict gRNA-recognizing sites in a target locus (exon 2 of rat *Tyr*). Three Alt-R® CRISPR-Cas9 crRNAs were chosen that match a 20-bp DNA sequence just upstream of the protospacer adjacent motif (PAM) (Fig. 1a): crRNA1, 5′-TTTCCAGGATTATGTAATAG-3′; crRNA2, 5′-GGACCACTATTACATAATCC-3′; and crRNA3, 5′-TTATGTAATAGTGGTCCCTC-3′. The three gRNAs overlapped each other and bound to the target sequence where a G to A mutation exists in an albino background. crRNA1 has already been used as Tyr-wild-crRNA in our previous study [18]. crRNAs were synthesized by Integrated DNA Technologies, Inc. (IDT; Coralville, IA, USA), and Alt-R® CRISPR-Cas9 tracrRNA was also purchased from IDT. Each reagent was dissolved in Opti-MEM® medium (Thermo Fisher Scientific Inc., Waltham, MA, USA) at a final concentration of 100 μM, and stored at −80 °C until use.

crRNA and tracrRNA were mixed in equimolar concentrations (30 μM of each) and allowed to anneal at room temperature for about 10 min. The resulting crRNA:tracrRNA duplex was known as a chimeric gRNA. For *i*-GONAD-based KI using an ssODN (designated ssODN for *Tyr*), custom DNA synthesis of ssODN was carried out by Macrogen (Seoul, South Korea). This comprised a 64 bp left arm and 65 bp right arm homologous to exon 2 of rat *Tyr* (including the mutated nucleotide A) (Fig. 1a). In this sequence, the normal nucleotide (G) at cDNA position 960 was included. Lyophilized ssODN was resuspended in Opti-MEM® medium at 10 μg/μL, then 2 μL was added to gRNA (6 μL) and 1 μL of recombinant Cas9 protein (10 μg/μL; IDT) in a 0.5-mL PCR tube for the preparation of RNP complex. Prior to *i*-GONAD-based KI, 1 μL of 0.2% Fast Green FCF (Nacalai Tesque Inc., Kyoto, Japan) was added to the mixture in a total volume of 10 μL. Final concentrations were: 30 μM gRNA, 1 μg/μL Cas9 protein, 2 μg/μL ssODN, and 0.02% Fast Green FCF.

For *i*-GONAD-based KI with KI-enhancing drugs, we used SCR7 (Xcess Biosciences, Inc., San Diego, CA, USA), EP Enhancer (IDT), AZT (Xcess Biosciences), L755 (Xcess Biosciences), RS-1 (Xcess Biosciences), and HDR Enhancer (IDT). These reagents were dissolved in dimethyl sulfoxide (Sigma-Aldrich, MI, USA) at a final concentration of 50 mM, and stored at −20 °C until use. Each reagent was then added to Opti-MEM® medium containing RNP complex, ssODN, and Fast Green FCF. The concentration and property of each reagent are shown in Table 1 and Additional file 1: S1, respectively.

### *i*-GONAD method

In this study, *i*-GONAD was conducted at E0.7 (at 16:00 on the day sperm was confirmed in the vagina). Prior to *i*-GONAD, pregnant females were anesthetized by intraperitoneally (IP) injecting the three combined anesthetics: medetomidine (0.75 mg/kg; Nippon Zenyaku Kogyo Co. Ltd., Fukushima, Japan), midazolam (4 mg/kg; Sandoz K.K., Tokyo, Japan), and butorphanol (5 mg/kg; Meiji Seika Pharma Co., Ltd., Tokyo, Japan).

*i*-GONAD was performed based on our protocols described by Gurumurthy et al. [17] and Takabayashi et al. [18]. Briefly, the ovary, oviduct, and part of the uterus were exposed by an incision made in the dorsal skin. Adipose tissue around the ovary was pinched by an Aorta-Klemme and anchored to prevent return of the exposed tissues. Approximately 1.5 µL of RNP or RNP with KI-enhancing drugs were injected into the oviductal lumen upstream of the ampulla using a glass micropipette connected to a silicon tube and a mouthpiece. The glass micropipette was made using an electric puller (Narishige Co. Ltd., Tokyo, Japan) and attaching mouth pipettes via aspirator tube assembly (Drummond Scientific Company, Broomall, PA, USA). After injection, the oviducts were covered with a piece of Kimwipes paper (Nippon Paper Crecia Co., Ltd., Tokyo, Japan) soaked in Dulbecco’s modified phosphate-buffered saline (DPBS), grasped in tweezer-type electrodes (Nepa Gene Co. Ltd., Chiba, Japan), and electroporated using a square-wave pulse generator NEPA21 (Nepa Gene Co. Ltd.). EP parameters were as follows: Poring pulse, 50 V, 5 ms pulse, 50 ms pulse interval, 3 pulses, 10% decay (± pulse orientation) and Transfer pulse, 10 V, 50 ms pulse, 50 ms pulse interval, 3 pulses, 40% decay (± pulse orientation). After in vivo EP, wet paper was removed from the oviduct, which was returned to its original position in the peritoneal cavity. The peritoneum and epidermis were sutured with suture thread and clips, respectively. Rats received the IP-administered medetomidine antagonist Antisedan (0.75 mg/kg; Nippon Zenyaku Kogyo Co. Ltd.) to recover from anesthesia, and were kept warm on a hotplate until they had recovered. They were then returned to the animal cages and housed as before until sacrifice.

### Analysis of CRISPR/Cas9-induced KI

*i*-GONAD-treated females were sacrificed at E14.5 to E16.5 under deep anesthesia using isoflurane (MSD Animal Health KK, Tokyo Japan). Fetuses were dissected from the uterus and immersed in DPBS. The presence or absence of pigmented eyes and the entire fetal morphology were observed under a dissecting microscope and photographed. Part of the tail was collected in a 0.2-mL tube and stored at −20 °C for genomic DNA extraction.

Genomic DNA was isolated from tail biopsies by incubating in 100 µL of 50 mM NaOH at 95 °C for 10 min. Then, 10 μL of 1 M Tris–HCl (pH 8.0) was added and mixed, and used as a template for PCR.

PCR was performed in a 20-μL reaction volume containing 1 µL of crude lysate DNA, 10 µL of 2 × PCR buffer for KOD FX, 0.4 mM dNTPs, 0.25 µM primer pairs (Tyr–F: 5′-GCTCAAGGTTTAGTTGGGTACT/Tyr–R: 5′-CTGGCTAGGTTTACTATCTCCTTG-3′), and 0.1 U KOD FX (TOYOBO, Osaka, Japan). The expected amplification product was 597 bp, containing *Tyr* spanning the mutated site (which might have been corrected by KI). PCR conditions were: initial denaturation at 94 °C for 3 min, followed by 33 cycles of 95 °C for 20 s, annealing at 57 °C for 30 s, and polymerization at 68 °C for 1 min, with a final extension at 68 °C for 5 min. PCR products (8 μL) were resolved on a 2% agarose gel and visualized by staining with ethidium bromide.

Direct sequencing was performed using the PCR products and the primer Tyr–F with a BigDye Terminator v3.1 Cycle Sequencing kit (Thermo Fisher Scientific Inc.), and then analyzed on an automated ABI PRISM 3100 DNA sequencer (Thermo Fisher Scientific Inc.).

### Evaluation of KI efficiency

The KI efficiency was determined from DNA sequencing results by dividing the number of fetuses judged to have undergone KI by the total number of fetuses recovered.

### Statistical analysis

To evaluate the effects of KI-enhancing drugs in *i*-GONAD-mediated KI, the chi-squared test was used to assess the significance of differences between groups treated with or without drugs. Differences with *p* < 0.05 were considered statistically significant.

## Supplementary Information

**Additional file 1**. **Table S1**: Summary of commercially available knock-in enhancers used in this study.

**Additional file 2**. **Fig. S1**: Comparison of efficiencies in samples showing KI, indels, mosaic mutations, or unedited samples, based on data shown in Table 1. *i*-GONAD was performed with crRNA1, 2, or 3, or a mixture of three gRNAs. Significant differences in KI efficiency, indel efficiency, and unedited efficiency were observed using a chi-square test, when samples treated in the presence of crRNA2 were compared with those treated in the presence of the other gRNAs. Abbreviations: 1, *i*-GONAD using crRNA1; 2, *i*-GONAD using crRNA2; 3, *i*-GONAD using crRNA3; 1+2+3, *i*-GONAD using a mixture of three gRNAs.

## Data Availability

Data is contained within the article or supplementary material.

## Abbreviations

AZT: Azidothymidine
Cas9: Clustered regularly interspaced palindromic repeats associated protein 9
CRISPR: Clustered regularly interspaced palindromic repeats
crRNA: CRISPR RNA
DSBs: Double-strand breaks
DPBS: Dulbecco’s modified phosphate-buffered saline
E: Embryonic day
EP: Electroporation
GM: Genetically modified
gRNA: Guide RNA
HDR: Homology-directed repair
HR: Homologous recombination
*i*-GONAD: Improved genome editing via oviductal nucleic acid delivery
indels: Insertion and deletion mutation
IP: Intraperitoneally
KI: Knock-in
KO: Knock-out
MMEJ: Microhomology-mediated end-joining
NHEJ: Non-homologous end joining
ODN: Oligodeoxynucleotide
PAM: Protospacer adjacent motif
PCR: Polymerase chain reaction
RNP: Ribonucleoprotein
RS-1: RAD51-stimulatory compound 1
ss: Single-stranded
tracrRNA: Trans-activating crRNA
Tyr: Tyrosinase
