# Supplementary material for: Modification of improved-genome editing via oviductal nucleic acids delivery (i-GONAD)-mediated knock-in in rats
Source: BMC Biotechnol. 2021 Nov 1;21:63. doi: 10.1186/s12896-021-00723-5 (PMC8561937; doi:10.1186/s12896-021-00723-5)
Supplement: Supplementary file 1 — Additional file 1. Table S1: Summary of commercially available knock-in enhancers used in this study. [file 12896_2021_723_MOESM1_ESM.docx]

Table S1. Summary of commercially available knock-in enhancers used in this study.

| Enhancer | Manufacturer | Note |
| --- | --- | --- |
| SCR7 | Xcess Biosciences | It functions as inhibitor of DNA ligase IV, which is repairing DSB *via* NHEJ [21]. It enhances HDR efficiency 4–5-fold in both human and mouse cell lines [23]. |
| Alt-R^Ⓡ^ Cas9 Electroporation Enhancer  (EP Enhancer) | IDT | It functions as an ssODN carrier that is computationally designed to be non-homologous to human, mouse, or rat genomes. |
| Azidothymidine (AZT) | Xcess Biosciences | Previously used as an anti-HIV drug that inhibits RT activity [20]. It enhances CRISPR-mediated KO *via* NHEJ in human iPSCs and other cell types, but decreases HDR efficiency 3-fold in ES cells [22]. |
| L755, 507  (L755) | Xcess Biosciences | Potent and selective β3 partial agonist. It enhances HDR efficiency 2-3-fold for large fragments and ~9-fold for point mutations in human iPSCs [22]. |
| RAD51-stimulatory compound 1  (RS-1) | Xcess Biosciences | It enhances HDR efficiency 3–6-fold *in vitro* and *in vivo* [24−26]. Also, it enhances CRISPR-mediated targeted KI efficiency 2-fold in bovine embryos [27]. |
| Alt-R^Ⓡ^ HDR Enhancer  (HDR enhancer) | IDT | Small molecule compound that promotes HDR in various cell lines. |

Abbreviations: DSB, DNA double-strand breaks; NHEJ, non-homologous end joining; ssODN, single-stranded oligodeoxynucleaotides; HDR, homology-directed repair; IDT, Integrated DNA Technologies, Inc.; RT, reverse transcriptase; CRISPR, clustered regularly interspaced short palindromic repeats; KO, knockout; iPSCs, induced pluripotent stem cells; KI, knock-in; ES, embryonic stem.
