# Supplementary material for: Modification of improved-genome editing via oviductal nucleic acids delivery (i-GONAD)-mediated knock-in in rats
Source: BMC Biotechnol. 2021 Nov 1;21:63. doi: 10.1186/s12896-021-00723-5 (PMC8561937; doi:10.1186/s12896-021-00723-5)
Supplement: Supplementary file 2 — Additional file 2. Fig. S1: Comparison of efficiencies in samples showing KI, indels, mosaic mutations, or unedited samples, based on data shown in Table 1. i-GONAD was performed with crRNA1, 2, or 3, or a mixture of three gRNAs. Significant differences in KI efficiency, indel efficiency, and unedited efficiency were observed using a chi-square test, when samples treated in the presence of crRNA2 were compared with those treated in the presence of the other gRNAs. Abbreviations: 1, i-GONAD using crRNA1; 2, i-GONAD using crRNA2; 3, i-GONAD using crRNA3; 1+2+3, i-GONAD using a mixture of three gRNAs. [file 12896_2021_723_MOESM2_ESM.pptx]

## Slide 1
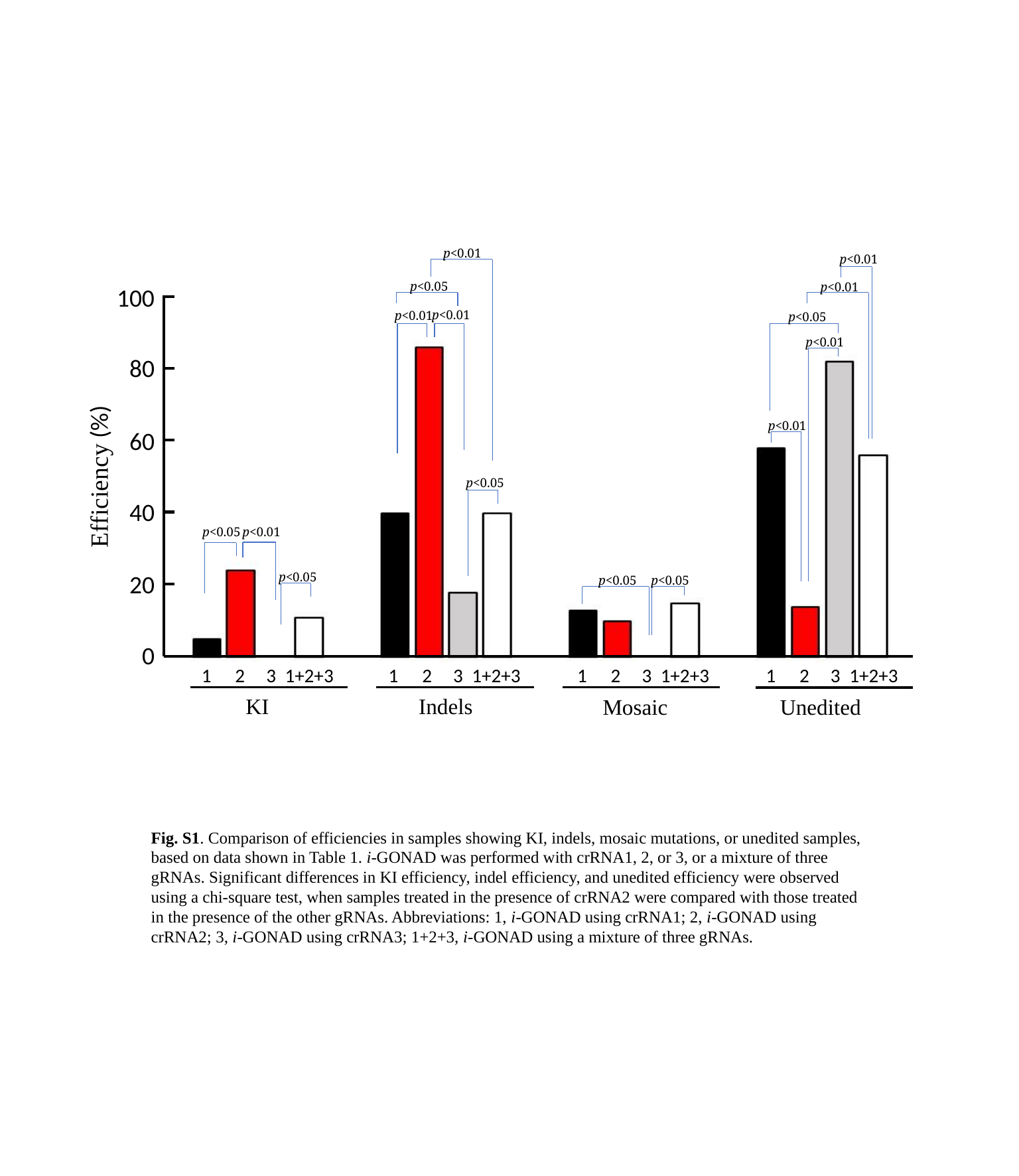

p<0.01
p<0.01
p<0.05
p<0.01
100
p<0.01
p<0.01
p<0.05
p<0.01
80
p<0.01
60
Efficiency (%)
p<0.05
40
p<0.05
p<0.01
20
p<0.05
p<0.05
p<0.05
0
1
2
3
1+2+3
1
2
3
1+2+3
1
2
3
1+2+3
1
2
3
1+2+3
KI
Indels
Mosaic
Unedited
Fig. S1. Comparison of efficiencies in samples showing KI, indels, mosaic mutations, or unedited samples, based on data shown in Table 1. i-GONAD was performed with crRNA1, 2, or 3, or a mixture of three gRNAs. Significant differences in KI efficiency, indel efficiency, and unedited efficiency were observed using a chi-square test, when samples treated in the presence of crRNA2 were compared with those treated in the presence of the other gRNAs. Abbreviations: 1, i-GONAD using crRNA1; 2, i-GONAD using crRNA2; 3, i-GONAD using crRNA3; 1+2+3, i-GONAD using a mixture of three gRNAs.
